# Updating “Angle-dependent spectral reflectance material dataset based on 945 nm time-of-flight camera measurements” with extended data to cover reflectance measurements mainly for vehicle varnish and moss rubber

**DOI:** 10.1016/j.dib.2023.109747

**Published:** 2023-10-31

**Authors:** David J. Ritter

**Affiliations:** Virtual Vehicle Research GmbH, Inffeldgasse 21a, Graz 8010, Austria

**Keywords:** Lidar, Lambertian, Reflectance, Spectral, Infrared, Material data, Time-of-flight, Angle-dependent

## Abstract

An updated dataset based on angle-dependent spectral reflectance measurements taken for an angle range of 0° to 80° with 10° incremental steps and pictures for most measurements of the whole dataset are presented in this article. It covers two new material types regarding the material classification scheme presented in the original paper [1]: vehicle varnish and moss rubber. Vehicle varnish samples are prepared on flat steel plates to enable proper angle-dependent acquisition of the data with the same angle adjustable measurement device already presented in the original paper.

The moss rubber material has a behavior similar to a Lambertian reflector and thus serves for diffuse reflectance measurement purposes. The extended dataset, including the original dataset, is published open access on the open repository Zenodo with record number 8322076 in a new version 1.1.1 [2], and currently contains 314 measurements with the addition of 70 new varnish and moss rubber measurements. The new version of the dataset further includes pictures of most of the measurements to depict how the data were acquired. The pictures enable material mapping such as the link between the measured data in the near infrared spectrum at a wavelength of λ=945nm and the picture taken in the optical spectrum.

Specifications Table

**Table:** 

Subject:	Material Physics
Specific subject area:	Angle-dependent spectral reflectance material measurements in the near infrared spectrum at a wavelength of λ=945nm taken by a time-of-flight camera.
Type of data:	Text files with two columns: reflectance *R*_λ=945_*_nm_* (%) and incidence angle *θ* (°). Table Pictures of measured samples
How the data were acquired:	There are no differences in the data acquisition methods compared to the original paper, therefore, it is the same as in the original data article.
Data format:	Raw
Description of data collection:	The intensity values of the time-of-flight camera are calibrated to Lambertian targets with well-defined reflectance values at 10%, 50% and 95% [1]. From the calibration process, a relation between intensity and reflectance of the Lambertian target is derived. Of the 300 frames taken by the time-of-flight camera with a resolution of 352×287 pixels, only the last 101 frames are considered. A line of 41 pixels perpendicular to the tilting angle of the image center at pixel row 145 of each of the frames, summing up to a total of 4141 datapoints is considered for evaluation. An outlier detection function of MATLAB with the Grubbs’ test method removes outlier datapoints. The average intensity is then used to calculate the reflectance value in the dataset.
Data source location:	Institution: Virtual Vehicle Research GmbH City: Graz Country: Austria
Data accessibility:	Repository name: Zenodo Data identification number: 8322076 Version: 1.1.1 Direct URL to data: https://doi.org/10.5281/zenodo.8322076
Related data article:	*D.J. Ritter, R. Rott, B. Schlager, S. Muckenhuber, S. Genser, M. Kirchengast, M. Hennecke, “Angle-dependent spectral reflectance material dataset based on 945 nm time-of-flight camera measurements”, Data in Brief, 48, p. 109031, 2023.*https://doi.org/10.1016/j.dib.2023.109031.
Related research article:	

## 1 Value of the Data

• The data extend the existing dataset with angle-dependent spectral reflectance measurements of a new material type: varnish of vehicles. The new data provides real measurements from flat varnish samples as direct measurements from vehicles are unfavorable due to the curvature of the vehicle parts with respect to the measurement device. The data consist of two different varnish types – glossy and mate – and a selection of ten color-s and four greyscales. The varnish measurements may enable more realistic modelling of the optical signature of vehicle varnish in IR within simulation.
• The data further include angle-dependent spectral reflectance measurements of the material moss rubber in ten colors and three greyscales. The material can serve diffuse reflectance measurement purposes as it exhibits a close to Lambertian reflector behavior.
• Provision of a basic reference for angle-dependent spectral reflectance values at a wavelength of λ=945nm in an angle range of 0° to 80° in incremental steps of 10°. This wavelength is very close to the typical range of common lidar wavelength of 830 nm to 940 nm which also has some exceptions such as 660 nm or 1550 nm [2]. This makes the use of this TOF camera representative for such wavelengths and enables comparison methodologies.
• Expansion of the dataset with pictures taken from the measured samples of the already published data and the extension to provide insight into how the data were taken for each measurement enabling a manual and simple material mapping of infrared to optical spectrum of the camera pictures.

## 2 Data Description

The presented data include different materials listed in the following:

• grounding of vehicle varnish
• vehicle varnish
• veneered furniture parts
• polymer foil
• moss rubber

The most important materials are the varnish of vehicles and the moss rubber. Both are additional material types (level 2) regarding the material classification presented in [3]. This material classification scheme is applied to the new measurements, and they are named accordingly, e.g., “*cover_varnish_green_glossy_1*” for varnish and, e.g., “*polymer_rubber_moss_blue_1*” for moss rubber. The angle-dependent spectral reflectance data are recorded for an incidence angle range of 0° to 80° in incremental steps of 10° at a wavelength of λ=945nm, which is close to the typical range of common lidar wavelengths [2], and are stored in text files (.txt) with two columns: incidence angle θ in degrees and spectral reflectance value $R_{\lambda }$ in percentage. As presented in [2], the spectral reflectance $R_{\lambda }$ is used to describe the radiometric response of a specific material. It is a function of the incidence angle θ and can be expressed as: $R_{\lambda }=f\left(\theta \right)\left[\%\right]$. The spectral reflectance values are given by the ratio of the measured intensity signal $I_{\lambda ,\theta }$ at wavelength λ, at an incidence angle θ and the intensity signal $I_{L,\lambda ,\theta =0{\;}^{\circ }}$ of a well-defined Lambertian reflector in nadir incidence ($\theta =0{\;}^{\circ }$) measured by the same measurement device, multiplied by the hemispherical spectral reflectance value $R_{L,\lambda }$ of the well-defined Lambertian reflector [1] at wavelength λ:

$$R_{\lambda }\left(\theta \right)=\frac{I_{\lambda ,\theta }}{I_{L,\lambda ,\theta =0{\;}^{\circ }}}*R_{L,\lambda }\left[\%\right]$$

**Fig. 1:**
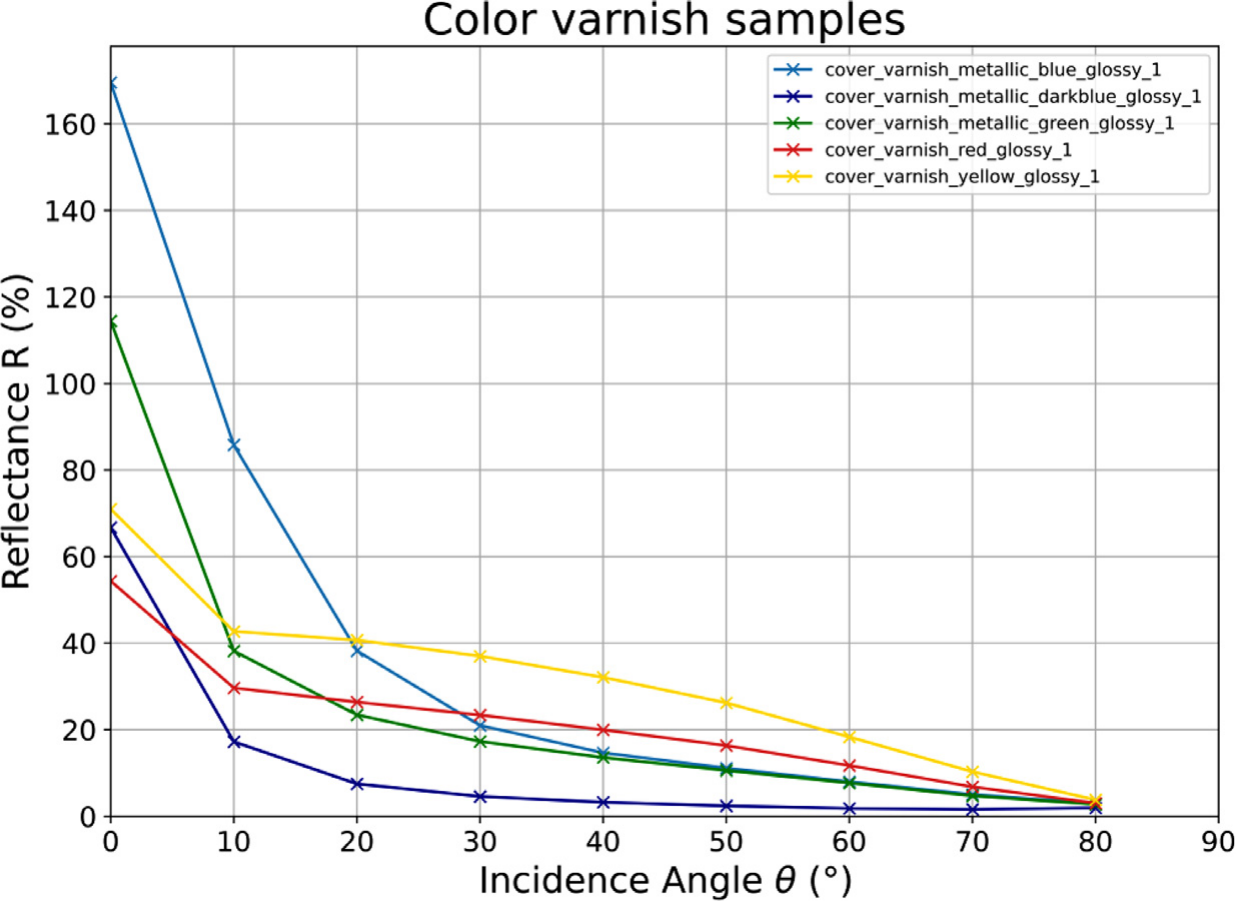
The measurement results taken from the vehicle varnish samples including five common colors of vehicles – blue, dark blue, green, red, yellow. The vehicle varnish samples red and yellow are of solid color, whereas blue, dark blue, and green are of metallic color. The left axis shows the spectral reflectance R given in percentage to a well-defined Lambertian reflector in nadir incidence ($\theta =0{\;}^{\circ }$) whereas the lower axis gives the incidence angle θ set at the measurement device in degrees.

**Fig. 2:**
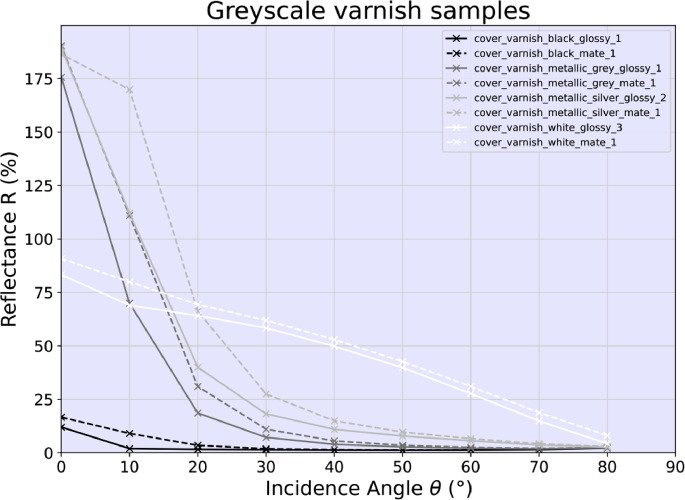
The measurement results taken from the vehicle varnish samples presented in common greyscales – black, grey, silver, white – and in two varnish types – mate and glossy. The greyscale vehicle varnish samples black and white are of solid color, whereas grey and silver are of metallic color The left axis shows the spectral reflectance R given in percentage to a well-defined Lambertian reflector in nadir incidence ($\theta =0{\;}^{\circ }$) whereas the lower axis gives the incidence angle θ set at the measurement device in degrees.

**Fig. 3:**
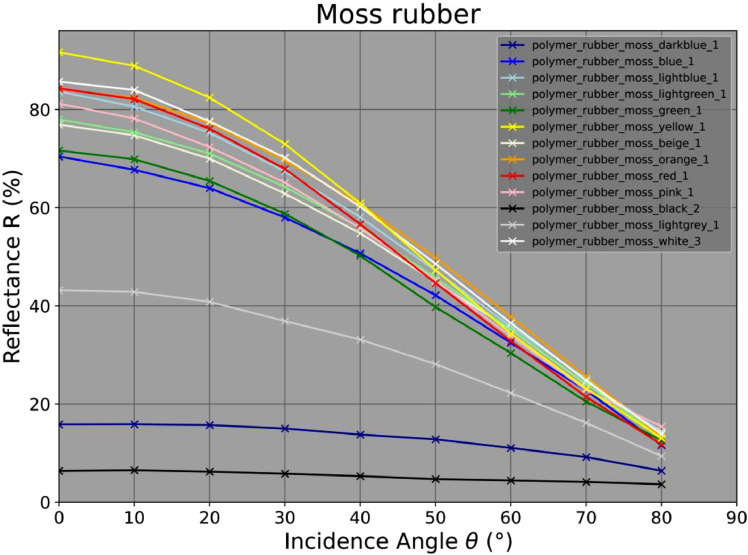
The spectral reflectance measurement results of all moss rubber materials. The left axis shows the spectral reflectance R given in percentage to a well-defined Lambertian reflector in nadir incidence ($\theta =0{\;}^{\circ }$) whereas the lower axis gives the incidence angle θ set at the measurement device in degrees.

Using hemispherical reflectance assumes that all wavelengths scatter the same way in a given direction. Since only one specific wavelength is used by the measurement setup, no iridescence, color change as a function of incidence and detection angle, is obtained. The five different color varnish samples – blue, dark blue, green, red, yellow – are shown in Fig. 1 whereas the different greyscale varnish samples – black, grey, silver, white – are presented in Fig. 2. The greyscale varnish measurements are taken from two different varnish types – glossy and mate. Fig. 3 contains the moss rubber measurement results in ten different colors to cover the range of the optical spectrum and three greyscales, and all moss rubber materials are listed in Table 2. All figures show the spectral reflectance $R_{\lambda =945\;nm}$ back to the illumination source.

The dataset is published on the repository Zenodo with the record number 8322076 in a new version 1.1.1 [4], including the new materials. In this version, the dataset currently contains 314 measurements and include not only one picture of the measurement setup as in [3], but pictures taken for almost each of the measurements. These pictures are essential to give insight into how the measurements were taken with respect to the environmental conditions, the placement and position of the measurement device over the material sample, and impression of the visual appearance of the material sample itself. This knowledge is not only crucial regarding the data acquisition process but further enables a possible manual and simple material mapping processes between the optical spectrum of the camera pictures and the NIR spectrum in which the measurement device operates.

## 3 Experimental Design, Materials and Methods

The angle-dependent spectral reflectance measurements are taken with the same measurement device presented in [3]. An Infineon IRS1125C TOF sensor operating at λ=945nm is cased in a ‘FusionSens Maxx GN8-1XNBA1 60 outdoor’ time-of-flight camera and mounted on a specific metal frame to facilitate manual adjustment of the incident angle. No adaptations to the measurement device have been made which would alter the measurement principle as well as the same measurement and evaluation scripts have been used to produce and evaluate the data. The pictures taken during the measurements are recorded with a mobile phone camera: Apple iPhone X. These pictures are recorded from no specific direction or distance as they only should facilitate the aforementioned insights. This allows to get impressions on the data acquisition but does not serve any high accuracy purposes.

**Fig. 4:**
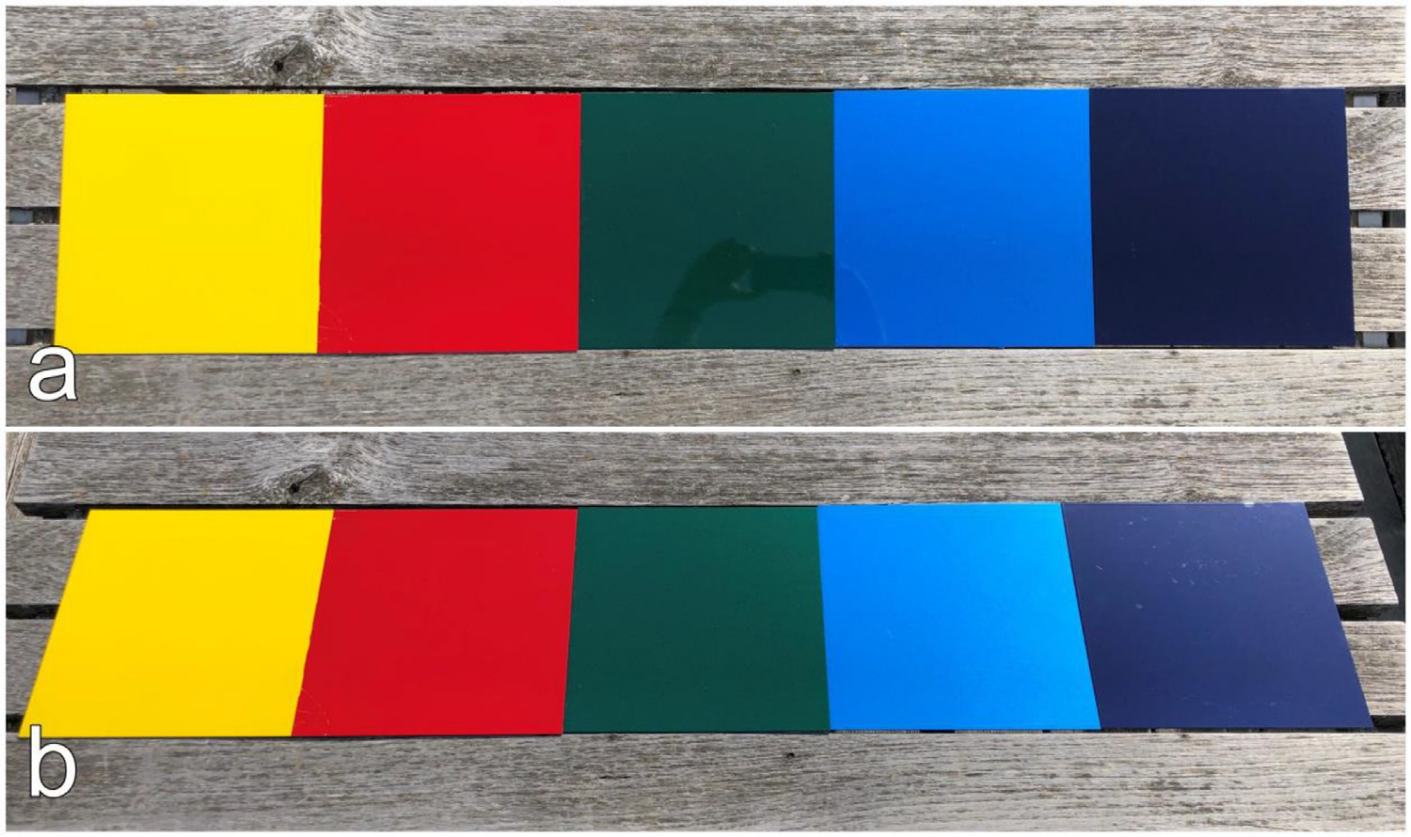
Colored vehicle varnish samples arranged in line to give an impression on their reflective behavior in the optical spectrum. The yellow and red samples are of solid color type whereas green, blue, and dark blue are of metallic type. (a) top view. (b) tilted view.

**Fig. 5:**
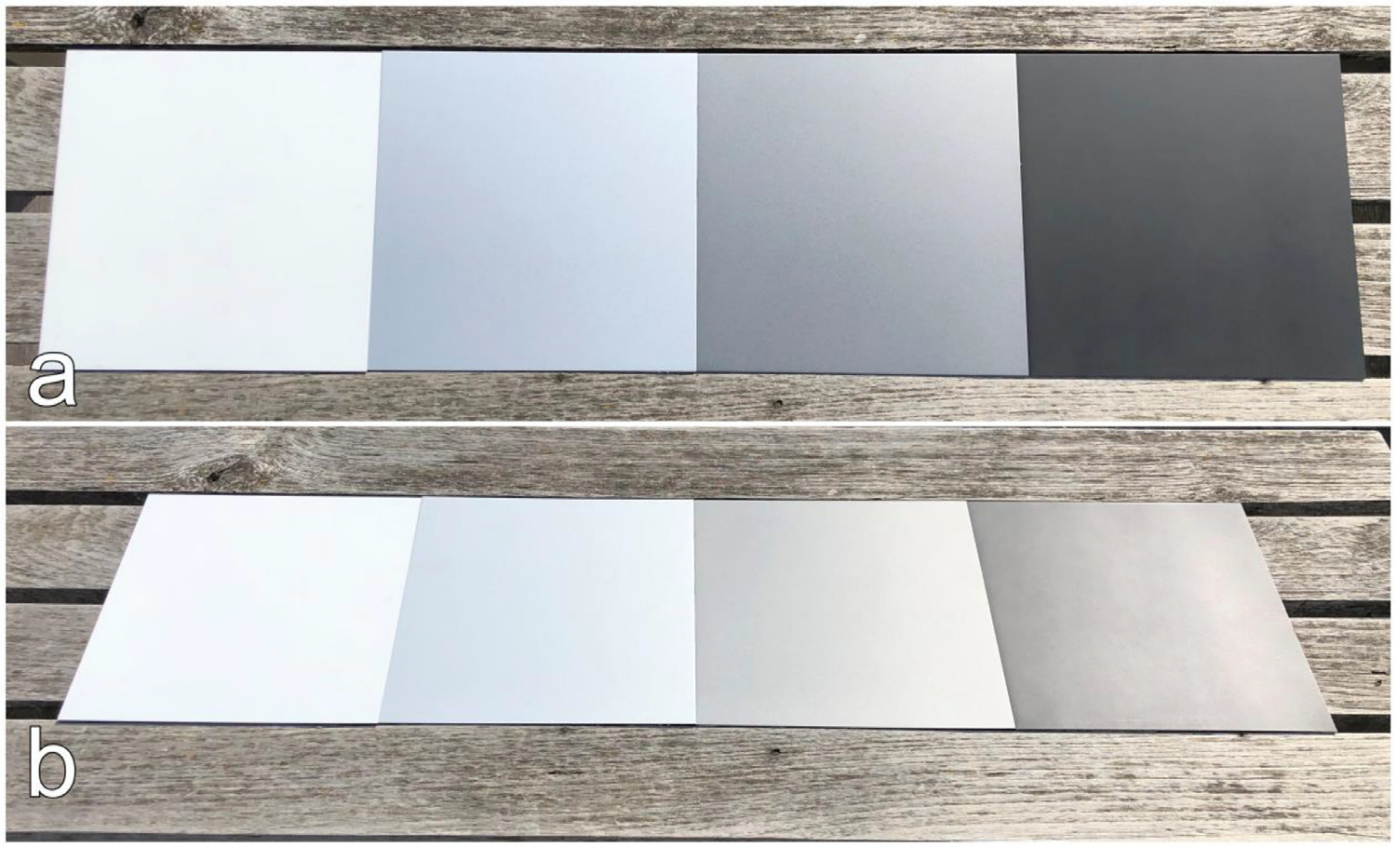
Mate vehicle varnish samples in different greyscale – white, silver (metallic), grey (metallic), black – arranged in line to give an impression on their reflective behavior in the optical spectrum. (a) top view. (b) tilted view.

**Fig. 6:**
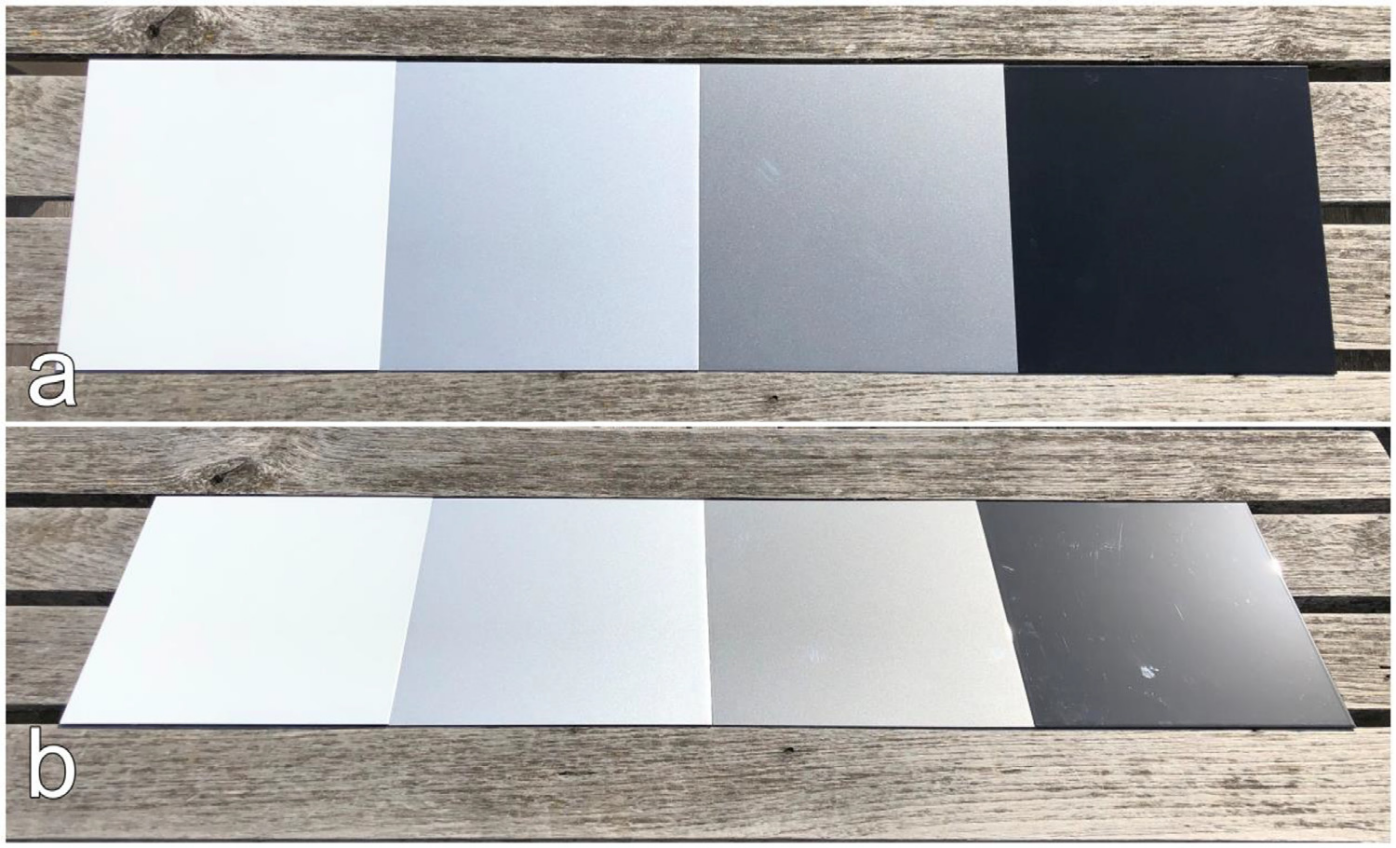
Glossy vehicle varnish samples in different greyscale – white, silver (metallic), grey (metallic), black – arranged in line to give an impression on their reflective behavior in the optical spectrum. (a) top view. (b) tilted view.

The vehicle varnish is a material of high importance in the automotive research field. Taking measurements of the varnish directly from a vehicle is impractical due to the curvature of the vehicle parts. The setting of the incidence angle at the measurement device described in [3] would not coincide with the angle of the measurement itself. To enable proper angle-dependent measurements with the measurement device, evenly flat varnish samples are prepared. The varnish is applied by a local paint shop^1^ onto ‘ST37 DC01 cold-rolled’ steel plates acquired from [5] with the dimensions of 250×250×1.5 mm. They are first coated with a grounding which can be chosen in three greyscales – white, grey, black – to support the color applied on top. Five colors and four greyscales are selected to cover the most common varnish types in everyday traffic situations. An overview of the 13 produced varnish samples is given in Table 1 where each varnish sample is indicated by a cross (x). There are two varnish types – mate and glossy – whereas glossy can be further divided into solid color and metallic color. In Fig. 4, Fig. 5, Fig. 6 the vehicle varnish samples are shown from two different perspectives: top view and tilted view.

**Table 1:** An overview of all evenly flat varnish samples produced in two different types on steel plates for angle-dependent spectral reflectance measurements. The varnish type glossy is further divided into solid color and metallic color. Each cross corresponds to a produced varnish sample by the local paint shop.

		Varnish type		
		Mate	Glossy	
			Solid	Metallic
Greyscale	White	×	×	
	Silver	×		×
	Grey	×		×
	Black	×	×	
Color	Dark blue			×
	Blue			×
	Green			×
	Red		×	
	Yellow		×

**Fig. 7:**
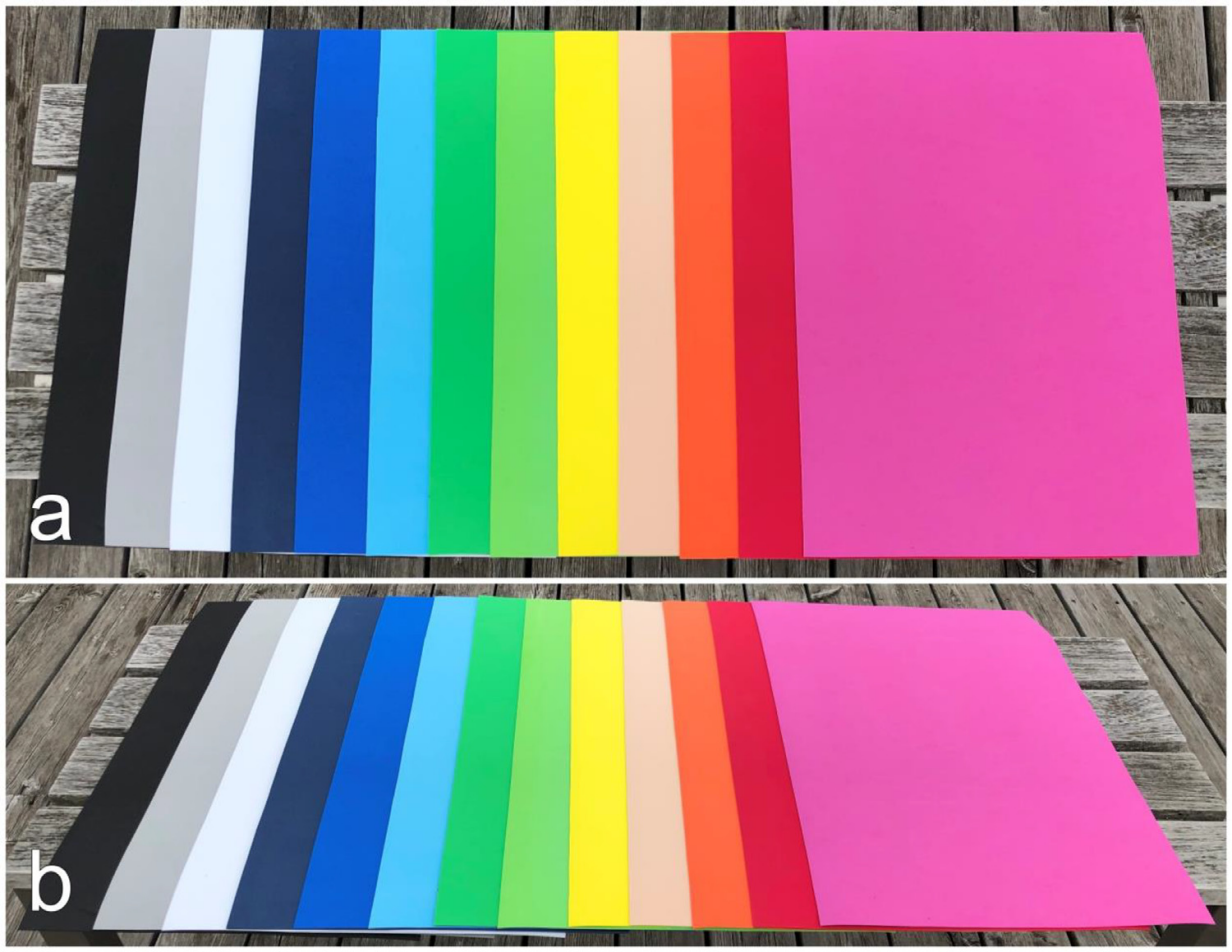
Three greyscale and ten color moss rubber samples arranged overlappingly in line to give an impression on their reflective behavior in the optical spectrum – black, grey, white, dark blue, blue, light blue, dark green, green, yellow, beige, orange, red, pink (from left to right). (a) top view. (b) tilted view.

The dataset is further extended by another material type, moss rubber. Within the classification scheme of [3] this material type (level 2) counts to the polymer material class (level 1). A selection of ten colors covering the range of the optical spectrum and three greyscales are chosen for taking angle-dependent spectral reflectance measurements. Some examples pictures are taken from the moss rubber samples and are shown in Fig. 7. The measured moss rubber samples acquired from an online shop [6] are listed in Table 2. The material is made of 50 % polyethylene, 45 % calcium carbonate, and 5 % other materials. Its surface is smooth, anti-skid, water-repellent and the optical look is mate.

**Table 2:** Greyscale and color moss rubber used for angle-dependent spectral reflectance measurement.

Greyscale	White
	Light grey
	Black
Color	Dark blue
	Blue
	Light blue
	Light green
	Green
	Yellow
	Beige
	Orange
	Red
	Pink

## Ethics Statement

This dataset generation campaign did not involve human subjects, animal experiments, or data collected from social media platforms.

## CRediT authorship contribution statement

**David J. Ritter:** Conceptualization, Software, Validation, Writing – original draft, Writing – review & editing, Investigation, Data curation, Visualization, Resources.

## Data Availability

Angle Dependent Spectral Reflectance Material Dataset based on 945 nm Time-of-Flight Camera Measurements (Original data) (Zenodo)

## Footnotes

1 LackundSpengler.at GmbH & Co KG
